# Multi-Armed 1,2,3-Selenadiazole and 1,2,3-Thiadiazole Benzene Derivatives as Novel Glyoxalase-I Inhibitors

**DOI:** 10.3390/molecules24183210

**Published:** 2019-09-04

**Authors:** Qosay A. Al-Balas, Mousa L. Al-Smadi, Mohammad A. Hassan, Ghazi A. Al Jabal, Ammar M. Almaaytah, Karem H. Alzoubi

**Affiliations:** 1 Department of Medicinal Chemistry and Pharmacognosy, Faculty of Pharmacy, Jordan University of Science and Technology, Irbid 22110, Jordan; 2 Department of Applied Chemical Sciences, Faculty of Science and Arts, Jordan University of Science and Technology, Irbid 22110, Jordan; 3 Department of Clinical Pharmacy, Faculty of Pharmacy, Jordan University of Science and Technology, Irbid 22110, Jordan

**Keywords:** Glyoxalase-I, Inhibition, Zinc Binding Feature, 1,2,3-Thiadiazole, 1,2,3-Selenadiazole

## Abstract

Glyoxalase-I (Glo-I) enzyme was established to be a valid target for anticancer drug design. It performs the essential detoxification step of harmful byproducts, especially methylglyoxal. A robust computer-aided drug design approach was used to design and validate a series of compounds with selenium or sulfur based heterorings. A series of in-house multi-armed 1,2,3-selenadiazole and 1,2,3-thiadiazole benzene derivatives were tested for their Glo-I inhibitory activity. Results showed that these compounds bind Glo-I active sites competitively with strong potential to inhibit this enzyme with IC_50_ values in micro-molar concentration. Docking poses revealed that these compounds interact with the zinc atom at the bottom of the active site, which plays an essential role in its viability.

## 1. Introduction

**Figure 1:**
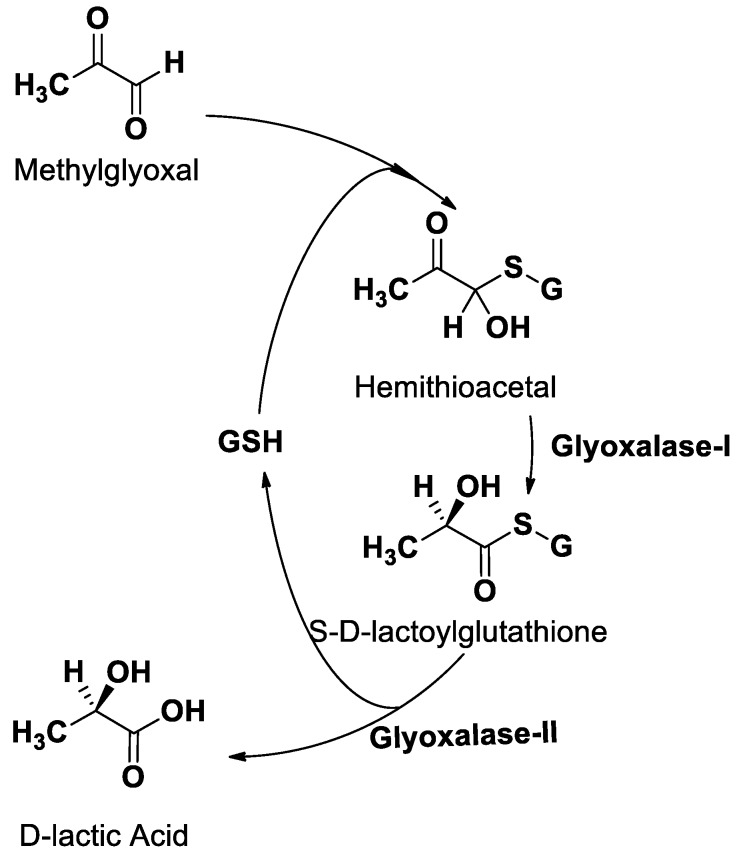
Detoxification of methylglyoxal by the glyoxalase system.

1,2,3-Thiadiazole and 1,2,3-Selenadiazole heterocycles, including their multiple ring derivatives, display diverse pharmacological activities. Their 4-alkyl or aryl substituted derivatives have demonstrated excellent activities as antiviral, anticancer, antibacterial, and anti-microbial agents [1]. On the other hand, aromatic and heteroaromatic compounds containing sulfur, selenium, and nitrogen atoms are useful substrates for the preparation of various heterocyclic systems [2]. The need for chemotherapeutic agents is increasing due to the limited abilities of the present drugs to cure and improve survival in many diseases [3]. The glyoxalase system, which consists of glyoxalase-I (Glo-I) and glyoxalase-II (Glo-II), is a metabolic pathway that is responsible for the detoxification of methylglyoxal (MG) by converting it to lactate in the presence of glutathione as a cofactor. Glo-I catalyzes the conversion of hemithioacetal (non-enzymatically formed) into S-d-Lactoylglutathione (SLG), and subsequently, Glo-II catalyzes the hydrolysis of SLG to a nontoxic d-lactic acid [4,5,6] (Figure 1). The MG is a highly reactive dicarbonyl compound that can be toxic to the cell [7,8]. Tumor cells are characterized by their elevated metabolic rate, resulting in the escalation of the sequential toxic metabolites, including MG and SLG [9,10]. Tumor cells counter that by intensifying the activity of both Glo-I and Glo-II [8,11,12,13] as reported in multiple cancers such as stomach cancer [14,15], murine fibrosarcoma [16], pancreatic cancerous tissues [17], colon cancer [18], prostate cancers [19], and endometrial cancer cells [20]. Inhibition of Glo-I enzyme in cancer cells will lead to the accumulation of MG, leading to cytotoxicity and, subsequently, self-destruction of the cancer cells [7,21,22]. These findings prove that the glyoxalase system could be used as a valid target for anticancer therapy.

For many decades, the glyoxalase system (particularly Glo-I) attracted the attention of researchers as a promising anticancer target [12,23]. For example, More et al. [24,25,26] proposed the first glutathione based Glo-I inhibitors. Natural products, on the other hand, such as coumarins [27], flavonoids [28], and anthocyanidins [29] have shown noticeable Glo-I inhibitory activity. Recently, we used in silico modeling to investigate the active site of the Glo-I enzyme and revealed that three main parts exist inside its active site; a highly polarized mouth, a zinc atom, and a deeply hydrophobic pocket. These particular features were useful when it came to searching for a commercially available Aldrich^CPR^ database ketol group [30] and also for designing a novel inhibitor of Glo-I enzyme possessing a thiazolecarboxylic acid moiety [31,32]. In the current study, molecular docking, using the LibDock protocol within discovery studio (DS 2017), of a series of multi-arm 1,2,3-selenadiazole and 1,2,3-thiadiazole benzene derivatives was carried out. The tested compounds were then assayed in vitro against the Glo-I enzyme, and Pearson product-moment correlation was done between in silico and in vitro results. 

## 2. Results and Discussion

**Figure 2:**
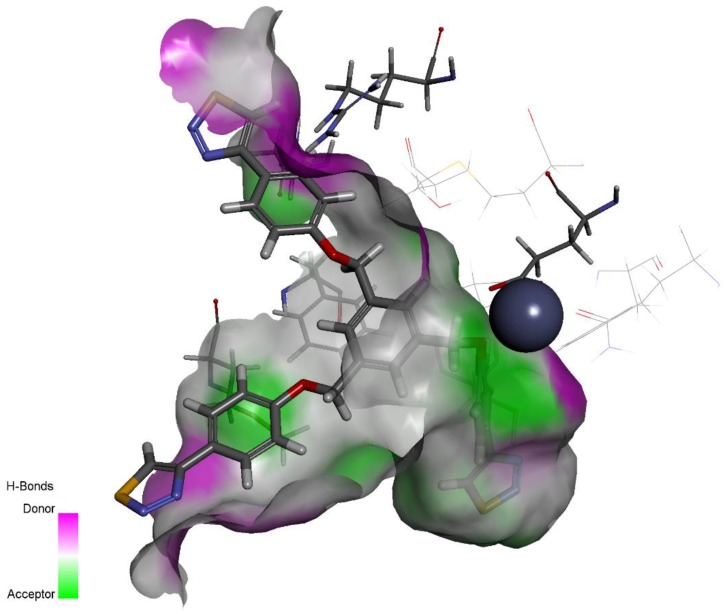

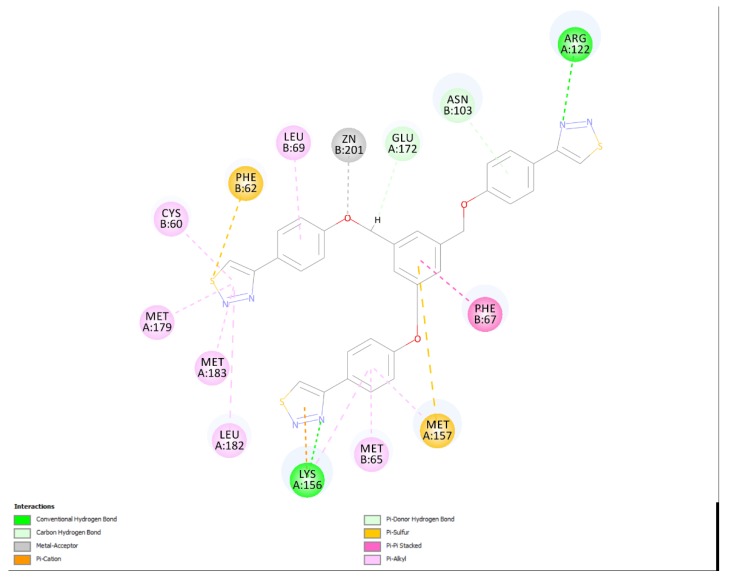
A docked pose of compound **8** with an IC_50_ = 3.0 µM inside the actives site. A three-dimensional representation of compound **8** showing its fitting inside the active site (Top). A two dimensional diagram showing the most important interactions with the active site amino acids: green, conventional hydrogen bond; sky blue, carbon hydrogen bond; grey, metal acceptor; orange, Pi-Carbon; light orange, pi-sulfur; purple, pi-pi stacked; light purple, pi-alkyl. (Bottom).

A series of twelve heterocyclic compounds containing multi-armed 1,2,3-selenadiazole and 1,2,3-thiadiazole benzene derivatives were tested against the Glo-I enzyme as described in the methods section, and the results obtained are presented in Table 1. All tested compounds showed appropriate activity with low IC_50_ values. In general, compounds containing di and tri methylene phenoxy spacers (5–8, IC_50_ = 3.0–23.6) showed higher activity than those counterparts without a methylene phenoxy spacer (1–4, IC_50_ = 32–64), which indicates the importance of the linker to generate a better fitting inside the active site. This fitting is mainly attributed to better flexibility of the compounds by filling the pockets optimally. Adding to this is the presence of an oxygen atom, which introduces a coordinate bond with the zinc atom within the active site. The oxygen atom is performing zinc chelation, which is normally accounted to be the major contributor of activity in Glo-I (Figure 2). Compounds containing 1,2,3-thiadiazole ring (**2**, **8**, **10**, and **12**) showed lower IC_50_ values (better activity) compared to their 1,2,3-selenadiazole analogues (**1**, **7**, **9**, and **11**). The opposite was observed in the case of compounds (**3** and **5**) such that those containing selenium hetero atoms gave higher IC_50_ values (lower activity) compared with those containing sulfur hetero atoms with the same number of arms (**4** and **6**). Based on these findings, an argument could be deduced that sulfur or selenium atoms are not crucial determinants for activity against the Glo-I enzyme when the compounds have lower molecular weight, and both are expected to perform weak hydrogen bond acceptor properties. The most active compound was found to be the tetra-armed 1,2,3-thiadiazole derivative (**10**), which has four sulfur hetero atoms and was found to be eight times more active than its selenium containing counterpart. This could be attributed to the importance of the size of the selenium atom when the active site is fully occupied by the tetra-armed moiety. Interestingly, this trend was repeated in the hexa-armed inhibitors where the sulfur analogue is approximately three times more active than the selenium analogue.

**Figure 3:**
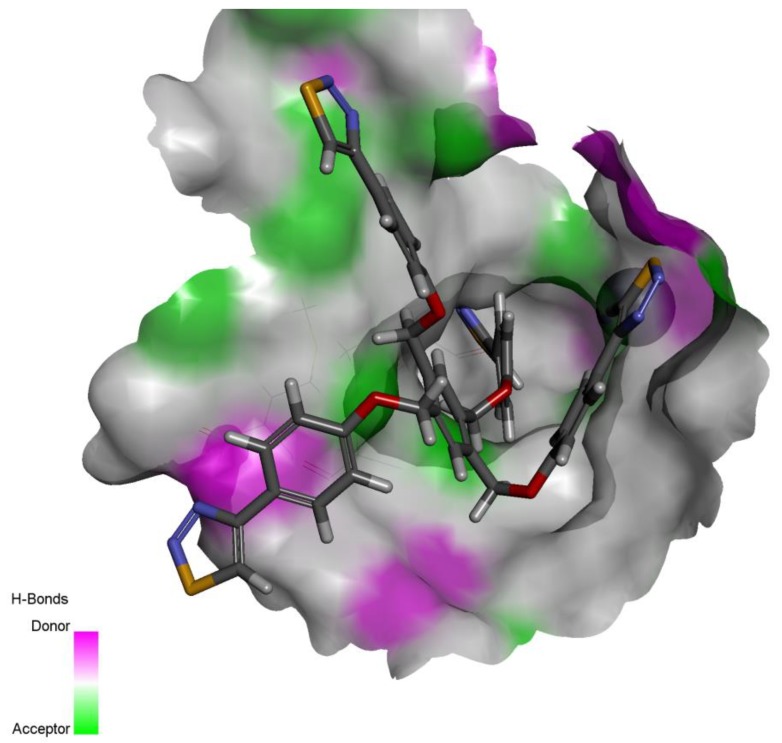

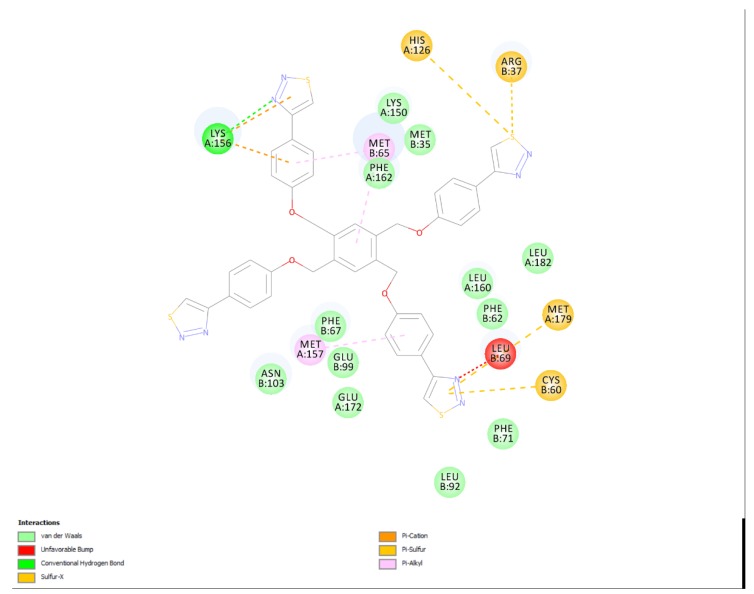
The most active compound **10** docked inside the active site of Glo-I (Top). A two dimensional simplified diagram of the binding pattern of compound **10**, which is showing the important interaction with Arg and His amino acids at the mouth of the active site (Bottom).

The most active compound **10** is shown to be filling the active site in addition to performing optimal obstruction of the mouth of the active site, a phenomenon called the umbrella effect in enzyme inhibition strategy (Figure 3).

### Correlation between the in silico and the in vitro results

**Figure 4:**
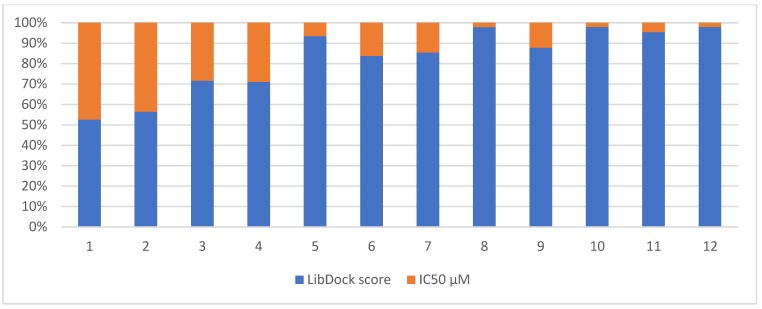
Column chart representation of the correlation between the LibDock score and the in vitro IC_50_ values as reported in Table 1.

The Pearson correlation coefficient was used to measure the linear correlation between two variables. Its values range from −1.0 to +1.0, with a score of −1.0 implying a strong negative correlation. The correlation coefficient (r) between the LibDock scores and the IC_50_ values was −0.76, indicating a strong negative correlation. Figure 4 illustrates the pattern of correlation between the computational and experimental scores. It is obvious that high scores are associated with low IC_50_ values in general, with some exceptions that are recognized to the limitation of in silico docking to rehearse the real behavior of the compounds. For example, compounds **8** and **10** were found in vitro to be the most active compounds; this is seen on Figure 4 as a very small orange area for the corresponding bars as an indication of low IC_50_. On the other hand, high LibDock scoring, which is seen for compounds 8 and 10 as a very large blue area that is correlated to a high LibDock score. On the opposite side, low activity means high IC_50_ (large orange area within the bar) and a low LibDock score (small blue area in the bar), which is noticeable for compounds **1** and **2** (Figure 4).

## 3. Materials and Methods

### 3.1. Computational Materials

ChemBioDraw Ultra 12.0 (PerkinElmer Inc., Waltham, MA, USA) was used to sketch the selected compounds. Discovery Studio (DS) 2017 (BIOVIA Software Inc., San Diego, CA, USA) was used to prepare the GLO-I crystal structure, while molecular docking was performed using the LibDock docking protocol within the DS GraphPad Prism (GraphPad Software Inc., CA, USA), which was used in calculating the percent enzyme inhibition and inhibitor IC_50_ values.

### 3.2. Experimental Materials

A total of twelve synthesized multi-arm benzene derivatives of 1,2,3-selenadiazole and 1,2,3-thiadiazole by *M.* Al-Smadi et al. [33,34,35] were selected for the present study. The *in vitro* inhibitory activity of the compounds against the human Glo-I enzyme was measured using double-beam UV-Vis spectrophotometer (Biotech Engineering Management Co. Ltd., UK). 

### 3.3. Computational Methods

#### 3.3.1. Ligand preparation

The investigated compounds were sketched using ChemBioDraw 12 and imported into DS to be subsequently converted into the corresponding three dimensional structures. The *prepare ligand* protocol within DS was utilized to generate the three dimensional structures, assign proper bond orders, and generate accessible tautomer and ionization states prior to virtual screening. Default parameters were used.

#### 3.3.2. Preparation of Glo-I Enzyme

The crystal structure of Glo-I in complex with N-hydroxypyridone derivative inhibitor (HPU) was retrieved from the Protein Data Bank (entry code 3W0T: resolution 1.35 Å) to serve as a structural model. *Protein Report* tool in DS was used to check the Glo-I structure for problems related to alternate conformations, missing loops, or incomplete residues. Then the crystal structure was cleaned using the *Prepare Protein* protocol in DS to fix such problems. The definition of the active site took into consideration the space ligands need in the docking process. 

#### 3.3.3. Molecular Docking

Molecular docking was performed using the LibDock algorithm within DS. LibDock is a high throughput docking algorithm that positions catalyst generated ligand conformations in the protein active site based on polar and nonpolar interaction sites (hotspots). The binding site of Glo-I was defined using the *Define and Edit Binding Site* tool within DS by a sphere of 16 Å radius.

#### 3.3.4. In vitro Enzyme Assay

The Glo-I inhibitory activity was determined as reported previously [31]. Briefly, human recombinant Glo-I (rhGlo-I), provided by R&D Systems^®^ Corporation, was reconstituted by dissolving 0.5 mg/mL of it in sterile, deionized water before storing at –70 °C. The tested compounds were dissolved in DMSO to 10 mM stock solution. The assay buffer was prepared by mixing 0.1 M sodium phosphate dibasic solution and 0.1 M sodium phosphate monobasic solution with a pH of 7.0–7.2. The substrate mixture was prepared by mixing a suitable volume of 0.1 M sodium phosphate assay buffer with 100 mM methylglyoxal solution and 100 mM reduced glutathione. Finally, the tested compounds were mixed with the assay buffer, substrate solution mixture, and the Glo-I enzyme in a cuvette at an appropriate tested concentration. Each compound was tested in triplicates at λ_max_ = 240 nm for 200 s at 25 °C.

#### 3.3.5. Correlation between the Docking Scores and in vitro IC_50_ Values

The Pearson Product Moment Correlation coefficient(r) was used to investigate how much the real and theoretical values of enzyme inhibition are related. It tested the linear correlation between two data sets using the Pearson formula.

## 4. Conclusions

A series of 12 compounds extracted from our in-house database with multi-armed 1,2,3-selenadiazole and 1,2,3-thiadiazole benzene derivatives have been tested in vitro for their capability of inhibiting Glo-I enzyme. A wide range of activities resulted with the most active compound (**10**, IC_50_= 2.4 µM), which showed superior inhibitory activities over the rest of the compounds. Interestingly, in silico studies were strongly correlated with the in vitro data, which encourages the use of this docking protocol to predict the activities of other compounds within commercial databases.

## Figures and Tables

**Table 1:** Enzyme inhibitory activities of a series of selected multi-arm 1,2,3-selenadiazole and 1,2,3-thiadiazole benzene derivatives (**1**–**12**).

Index	Chemical Structure	LibDock Score	IC_50_ µM
1	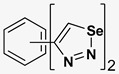	71.218	64.00
2	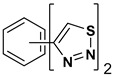	76.639	61.10 ± 9.1
3	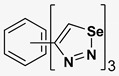	77.4038	32.10 ± 5.5
4	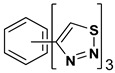	83.3944	34.01 ± 1.9
5	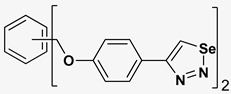	109.94	7.90 ± 1.8
6	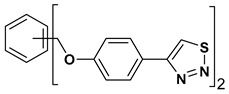	117.882	23.60 ± 1.7
7	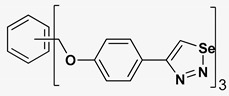	135.237	23.06 ± 1.6
8	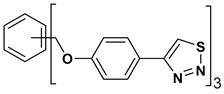	143.245	3.00 ± 0.15
9	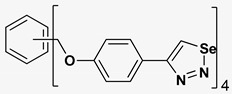	139.985	19.53 ± 2.0
10	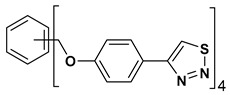	119.431	2.40 ± 0.13
11	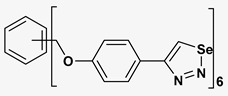	173.358	8.30 ± 0.56
12	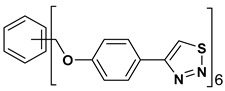	133.129	2.67 ± 0.31
